# How experienced wound care nurses conceptualize what to do in pressure injury management

**DOI:** 10.1186/s12912-023-01364-z

**Published:** 2023-06-05

**Authors:** Ye-Na Lee, Sung Ok Chang

**Affiliations:** 1 Department of Nursing, The University of Suwon, Hwaseong, Republic of Korea; 2 College of Nursing and BK21 FOUR R&E Center for Learning Health Systems, Korea University, 145, Anam-Ro, Seongbuk-Gu, Seoul, 02841 Republic of Korea

**Keywords:** Pressure injury, Wound, Phenomenography, Wound care nurse education

## Abstract

**Background:**

Conceptual understanding of the perceptions that wound care nurses use to determine how to manage pressure injuries may provide information for improving their pressure injury care competency. The aim of this study is to explore and describe the way wound care nurses experience and perceive pressure injury management.

**Methods:**

A qualitative, phenomenographic approach, a method designed to explore the different ways in which people comprehend a phenomenon and develop a practical knowledge-based framework, was used in this study. Semi-structured interviews were used for data collection with twenty wound care nurses. All participants were female with a mean age of 38.0, mean total clinical experience of 15.2 years and mean clinical experience as wound care nurse of 7.7 years. The eight steps of qualitative data analysis for a phenomenographic study were employed to develop an understanding of participants’ experience of pressure injury management.

**Results:**

The analysis resulted in an assessment domain and an intervention domain, each containing three descriptive categories based on five identified conceptions. The categories were as follows: “comparison”, “consideration”, and “monitoring” in assessment, and “creation”, “conversation” and “judgement” in intervention.

**Conclusions:**

This study has created a framework for understanding pressure injury management based on practical knowledge. This framework of the nurses’ pressure injury care reflected the need for an awareness of a harmonious approach to patients and wounds. There is a pattern of transcending a reliance on only theoretical knowledge, and this key factor in the framework should be considered when developing education programs and tools for improving nurse pressure injury care competency and patient safety.

## Background

The occurrence of pressure injuries (PIs) leads to economic losses caused by extended hospitalization and unnecessary increased medical costs for management [1–4]. There are also risks of complications, such as infections, which can increase when the condition is not properly treated through accurate and early assessment [5, 6]. The risk of death from sepsis also increases when the condition is not treated [1]. Therefore, PIs are becoming increasingly recognized as a patient safety issue across the world [7]. As wound-related issues are taken more seriously, nurses capable of professional PI care are increasingly in demand.

To meet this need, wound care nurses have increased, and the scope and roles are expanding [8, 9]. In addition, to enhance the overall PI care capacity of hospitals, PI education is emphasized to improve not only the competency of new wound care nurses but the competency of clinical nurses as a whole.

Clinical nurses have learned fundamental PI management, including anatomical and physiological knowledge related to skin and wounds and basic wound care according to their undergraduate curricula. After becoming clinical nurses they are provided with PI education both through continuing education and the PI guidelines formulated on the basis of evidence on the prevention and treatment of PI, in addition to regularly updated nursing intervention guidelines [10]. However, their continuing education has focused mostly on theoretical knowledge and been limited for developing nurses’ problem-solving skills and ability to apply knowledge in practice during actual PI care. Additionally, the clinical guidelines of PI care have primarily focused on the prevention rather than the treatment of PI, and the vast amount of content provided has hindered its practical use by clinical nurses as reference [11].

Although comprehensive knowledge as well as common sense are the basis of the decision-making process, it has been emphasized that practical knowledge is the most influential factor in wound management [12]. Several researchers have noted that, for nurses, when making decisions about wound management and expert decision-making in general, the hardest and most important part is the professional ability to focus on the most important aspects of the situation [13–15]. Such ability is nurses’ intuition of identifying the essence of the problematic situations, acquired through rich practical experiences [16]. Many previous studies have emphasized the importance of such practical knowledge in the management of PI [13–16].

However, according to previous studies, a clear gap exists between the guidelines and actual clinical practice, emphasizing the effectiveness of implementing clinical practical knowledge as a measure to reduce this gap [10, 11]. However, many tools and educational programs used in current clinical practice are based on theoretical guidelines and modified to apply to a hospital or department basis, resulting in limitations to reflect individual clinical situations. For example, a meta-analysis of the Braden Scale revealed that it had moderate predictive validity with good sensitivity and low specificity in critically ill adult patients [17]. Therefore, the studies recommend either the further development and modification of this scale or the generation of a new tool of higher predictive power for use in ICU populations who have a higher risk of PI. Accurate and valid assessment of PI is important to identify high-risk patients and to provide appropriate PI management [17].

In order to be effective, PI education for new wound care nurses and clinical nurses, should implement such practical-knowledge-based education. Practical-knowledge-based education would allow nurses to independently assess the situation and make decisions on patients’ health issues based not only on their skills, but also their professional knowledge [18]. To achieve this requires a method of identifying the structure of PI management based on the individual experiences of wound care nurses and the resulting understandings they have acquired. This study was conducted to identify the clinical experiences of wound care nurses in PI assessment and intervention, the structure of PI assessment and intervention tasks developed by wound care nurses through their clinical experience, and the wound care nurses’ awareness of tasks related to PI assessment and intervention. In this way, the study can establish a practical knowledge set for incorporation into education and training systems.

Accordingly, the present study aimed to identify how experienced wound care nurses know what to do in PI management. The results also explored the experiences of wound care nurses in their clinical practice of PI assessment and intervention, which serves as the base resource that reflects rich clinical experience. In this way the study contributes to the development of efficient PI education and training applicable to nurses’ clinical practice, thereby reducing the gap between knowledge and clinical nursing.

## Methods

### Design

This study applied phenomenography, which examines not the phenomenon itself, but the individual experiences of the phenomenon, and that focuses on and describes the difference between individuals in their experiences [19]. Phenomenography is used in the field of education to apply individual awareness of a phenomenon to the associated field of study [20, 21]. The phenomenography method is used to identify the different qualitative experiences people have of a certain phenomenon [19, 20]. This study by phenomenography focused on how different nurses viewed their awareness and how their awareness is related to the PI care process, which is in line with the focus on variation and how those variations are connected.

### Participants

Participants were purposively selected to maximize variation and ensure data saturation. In a phenomenography study, the sample size should be between 10 and 30, and, in this study, saturation was confirmed in the data collected from twenty wound care nurses. The inclusion criteria were that the participants had completed the Fellowship of Korean Wound Academy and had been working as wound care nurses for 3 years or more. According to Benner’s theory, the first three years of work experience is the period during which one becomes accustomed to a duty, and one’s problem-solving efficiency improves [22]. All 20 participants were female with a mean age of 38.0 years (± 3.9 standard deviation [SD]). 14 were master’s graduates, and 6 were doctoral graduates. The mean extent of total clinical experience was 15.2 years (± 4.6 SD) and the mean extent of clinical experience as a wound care nurse was 7.7 years (± 3.6 SD).

### Ethical consideration

This study was conducted after obtaining approval from the institutional review boards of Korea University Guro Hospital (No. KUGH17188-001). The study has been performed in accordance with the ethical standards as laid down in the 1964 Declaration of Helsinki and its later amendments or comparable ethical standards [23]. All interviewees were informed of the study purpose, methods and recording of interviews and were recruited only after they had agreed to participate and provided written consent. To ensure their voluntary participation, they were also informed of their right to withdraw their participation at any time during the study. Additionally, they were compensated for their participation as per ethical considerations. The names of the participants in the interview records were replaced with symbols and the interview records and transcripts were stored in a password-protected computer to ensure no unauthorized access to the data.

### Data collection

The study data were collected from November 2017 to June 2018. The interview session duration was approximately one hour per participant and the questions posed were as follows: “How and based on what do you assess PIs in clinical practice?” and “How and based on what do you treat PIs in clinical practice?”.

### Data analysis

The results of the phenomenography study are described by category and are comprised of an outcome space that indicates the relationship of the result with each category [20]. The conceptualization of experience is identified by determining categories and outcome spaces [19, 20, 24].

In this study, the interview data collected in Korean were translated into English and then analyzed. The translation used forward-translations and back-translation steps to achieve not only linguistic/literal equivalence but also cross-cultural and conceptual equivalence.

**Fig. 1:**
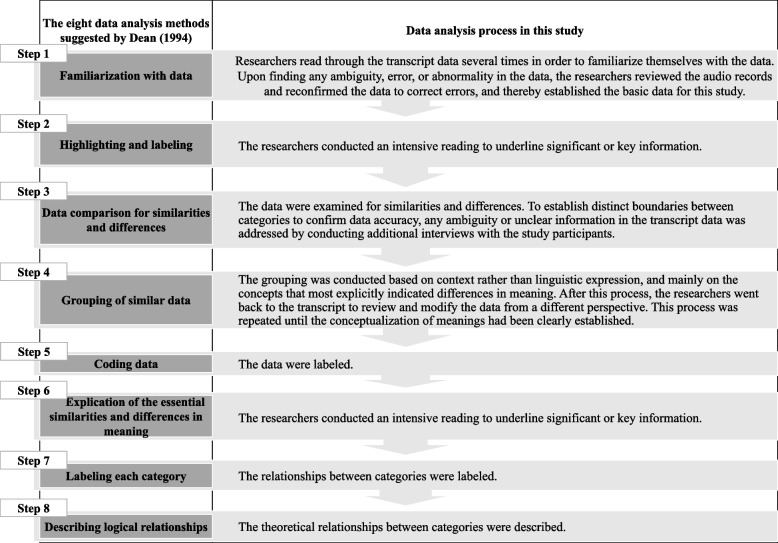
The Process of Data Analysis

Next, the data analysis methods suggested by Dean [24] were applied: familiarization with data, highlighting and labeling, data comparison for similarities and differences, the grouping of similar data, coding data, explication of the essential similarities and differences in meaning, labeling each category, and describing logical relationships (Fig. 1).

### Categories determination

In this study, one to six of the eight data analysis methods suggested by Dean [24] were used to determine the categories of wound management experience conceptualization by wound care nurses.

### Composition of outcome space

The composition of the outcome space refers to the internal relationships and hierarchical levels within each category [24]. When applying this approach, to structure the outcome space researchers may use methods based on theories or academic perspectives in the relevant fields. In this study, researchers identified the categories comprising the analyzed experience conceptualization and suggested the relationships between them. The outcome space was determined through the analysis steps 7 and 8 of Dean’s data analysis methods (Fig. 1) [24].

### Trustworthiness

To retain the study’s neutrality, the interviewer commenced without any intention of reaching a particular conclusion or proving a particular point and attempted to remain faithful to the interview data. Moreover, to minimize any bias or prejudice, a semi-structured questionnaire was used for the interviews to prevent the researcher’s bias from impacting the statements of the interviewees [25]. During the interview or analysis, any ambiguous or unclear parts of interviewee statements were clarified by follow-up questions. Two researchers analyzed the data after each interview and verified the meanings of the content and the categories [26]. The records of the interviews and results of the analyses were then sent to the interviewees via email or phone to ensure consistency between the descriptions written by the researchers in their analyses and the actual experiences of the study interviewees themselves [26].

## Results

Each perception of PI management is identified as assessment or intervention and discussed.

### How experienced wound care nurses conceptualize what to do in PI assessment

#### Category A1. Focusing on what caused the condition of the wound

Participants emphasized that they need to identify the cause of the wound accurately. Accordingly, participants should distinguish between PIs and other conditions such as incontinence-associated dermatitis, skin laceration, and moisture-associated skin damage.

*“An injury caused by friction is not considered a PI. Therefore, it is important to check the origin of an injury carefully to determine whether the injury site is actually impacted by pressure. In operation rooms, they occur in unusual sites, even sites lacking bone-prominence. This is why we learn the position of a patient in an operating room through reviewing the chart.” [Participant 13].*

#### Category A2. Interpreting by the sequential application of theoretical and practical knowledge

Participants evaluate PIs by identifying wound characteristics using the classification system suggested by the guidelines or theory. If there is any confusion, the specific experiences the nurses have had of each stage are compared to the current case to make the evaluation. Such knowledge gained from experience of success or failure in PI management is practical knowledge.

*“I learned that stage 2 involves dermis exposure and stage 3 involves fat. But sometimes it is hard to tell advanced Stage 2 from Stage 3 based on only such knowledge. What I do to distinguish stage 2 is to subtly scratch the injury with a blade. As dermis is of limited thickness and subcutaneous blood supply is low, in most cases the injury does not bleed. So, bleeding distinguishes between Stage 2 and 3.” [Participant 7].*

#### Category A3. Comparing the risk of aggravation and healing potential

Participants also assess whether the wound bed is covered with slough or necrotic tissue, whether the surrounding skin is red, and whether the tissue is healthy. They evaluate PIs by considering advanced aspects of the condition, such as how long it will take to heal. They emphasized that the assessment should be made considering the risk of aggravation and the healing potential.

*“I think we need to assess differently between superficial and deep wounds at Stage 2. This is important is because the target healing period is longer at deep Stage 2 than superficial stage 2, and this difference affects the direction and purpose of treatment. I assess the shape of the fat even within Stage 3. One form of fat has vesicles covered by a capsule like a peeled orange and the other form is loose in form but is not yet slough. The two cases would develop into different results, so they require different assessment.” [Participant 15].*

#### Category A4. Identifying the possibility of improving the potential for healing

Participants assess PIs at the same stage differently, considering individual patient characteristics such as age, pre-existing diseases, skin color, and skin thickness by location. They said a PI assessment needs to include a patient-oriented assessment comprehensive in scope, including the patient’s diseases, nutritional status and needs.

*“Even the same wound can have different results depending on patient characteristics such as age, and pre-existing diseases and conditions. This must be considered in the assessment as well. Accordingly, it may not be clinically correct to classify wounds as the same Stage 1 PI by simply assessing whether or not the wound is non-blanching erythema. Erythema affects different results based on the individual patient characteristics, and, therefore, different treatment methods should be considered.” [Participant 2].*

#### Category A5. Monitoring within the healing time frame

Participants not only perform an early assessment but also continuously assesses and record the status of a PI whenever dressing the wound. This category reflects the need for continuous and repetitive observations and checking-up on the development of exudation or signs of infection at intervals to adjust treatment methods accordingly.

*“Deep tissue injury (DTI) is hard to identify as those with an increased international normalized ratio (INR) have petechiae all over the body. It can be hard to tell whether there has been pressure on the site or not. So, when we wait and see how the condition develops, demarcation signs appear, it turns out to be DTI indeed. This is why DTI needs to be evaluated on a constant basis.” [Participant 10].*

### How experienced wound care nurses conceptualize what to do in PI interventions

#### Category I1. Strengthen the recovery ability of skin

Participants thoroughly consider how to improve support of the surface better and use products or instruments to control the temperature and humidity of the surrounding skin to improve tissue perfusion to recover damaged tissue. This category emphasizes efforts to improve the durability of the PI and the surrounding skin to let the skin naturally recover.

*“If the skin around the wound is dry, I apply moisturizers to help the wound heal. Instead of focusing only on the wound, I first apply sufficient moisturizer to surrounding skin to address the dryness and then apply the foam or ointment to the wound.” [Participant 17].*

#### Category I2. Creating an environment to maximize healing potential

Participants manage the hygiene of the patient and educate a caregiver or ward nurse about the importance of PI management. This category reflects the view that creating a proper therapeutic environment promotes healing. Moreover, the effort of medical staff and continuous support and care of other individuals are required for a good treatment result.

*“You can’t just ask the caregiver to change the position. You must explain and show directly about the appropriate position change for the site of the PI, and the caregiver should be guided to check with his or her hand to confirm the pressure is not loaded to the PI site.” [Participant 1].*

#### Category I3. Increasing positive signs of healing

When participants change the dressing, they assess the state of PI healing and expect positive signs, such as a reduced injury size or a decreased amount of exudation. This category is to ensure that the current treatment method yields positive outcomes and to maintain it or maximize the positive effects of fast healing.

*“In Stage 2, re-epithelization is important. You must ensure the edge of the wound is not dry and is well covered. And if it is confirmed, you can expect a better outcome. Similarly, in Stage 1, the skin color returns to normal in the process of healing. I perform more frequent check-ups on the status of a PI at Stage 1 than at the other stages.” [Participant 9].*

#### Category I4. Focusing on the interaction of dying cells with living cells

When choosing a treatment method to heal a wound, wound care nurses constantly think about priorities, while considering the impact on wound healing. In other words, participants constantly interact both with the cells regenerating within the injury and those dying. An integrated understanding of why some cells are alive and why some are dying helps the participants to promptly detect the occurrence of PI complications, such as infections, and prevent deterioration in advance through dressing, such as with antiseptics, antimicrobial medicines.

*“Many nurses are concerned whether they can use povidone iodine, which is toxic to normal cells. I think that if there are any signs of infection, such as malodor and slough, they should be dealt with first using iodine. You have to pass the infection stage before you can move on to the proliferation stage. You need to calculate what the gain is and what the loss is” [Participant 18].*

#### Category I5. Determining what I can and cannot do

Throughout treatment, participants adjust the care plan according to the monitoring results. When doing this, they need to properly determine the scope of their capability, thinking about what they can and cannot do as a wound care nurse including not only clinical decision-making but also privileging and credentialing. Proper judgment in this category can facilitate and accelerate the healing of PIs through an effective use of resources and collaboration.

*“PI is healed by the combination of every clinician’s effort. PIs will not heal no matter how hard I attempt a good treatment if the patients are not well-nourished or have poor circulation. Therefore, we need to consult with a nutritionist and a cardiovascular center. A PI is not something that can be addressed by a wound care nurse alone.” [participant 5].*

## Discussion

**Fig. 2:**
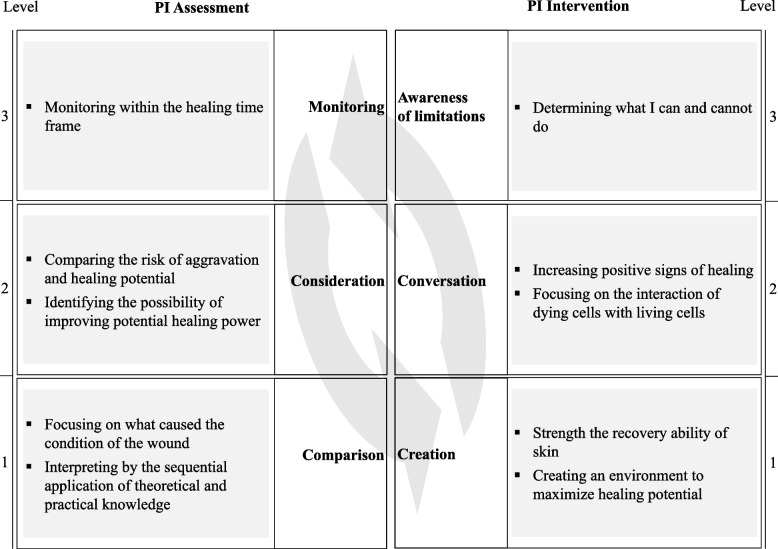
Outcome Space of PI Management

This study identifies the conceptualization of wound management experience by wound care nurses (Fig. 2, Table 1). The five categories of PI assessment obtained from the conceptualization are hierarchically structured from level 1 to level 3: Comparison, Consideration, and Monitoring. The five categories of PI intervention obtained from the conceptualization are hierarchically structured from level 1 to level 3: Creation, Conversation, and Awareness of Limitation.

**Table 1:** Outcome space of PI management

	Level	Focus	Purpose	Major activities
Assessment of PI	1	Comparison	To distinguish from other wounds and assess exactly using theoretical and practical knowledge	Identification of patient medical history relating to wounds and comparison of the characteristics of each stage as prescribed in guidelines and from experience
	2	Consideration	To identify not only the surface condition but also the potential power of patients when making a treatment plan	Concerning all factors that affect the treatment of PIs and evaluate which factors can be improved
	3	Monitoring	To identify changes in the PI and monitor progress against predictions and adjust the direction of treatment accordingly	Nursing activities that document and photograph the PI after assessment for each dressing and suggest that treatment plans and injury management methods may constantly change according to the evolving status
Intervention of PI	1	Creation	To aid self-healing, involving the management of both internal and external environmental aspects	Interventions for humidity, dryness, and pressure on the skin that affect skin durability. Interventions for hygiene management and education for the patient, caregivers, and ward nurses
	2	Conversation	To observe changes in the injury status to maintain positive factors for healing and eliminate negative factors for prevention purposes, thereby having all relevant factors for facilitating the treatment	Daily monitoring of epithelization, increase in tissue granulation, size reduction, etc. And preventing deterioration by treating infection
	3	Awareness of limitations	To identify one’s capabilities and limits, and to request support and resources when needed	Collaborate with other departments or medical teams for a multi-disciplinary approach

The necessity of developing an effective framework for improving the competency of nurses in PI care is apparent. A training frame is a contained experience of how professional wound care nurses actually solve problems and is designed to improve the confidence of clinical nurses in realistic PI care situations. This study provides a conceptualized framework based on the flow of perceptions that have emerged from the PI care experiences of wound care nurses. The study of decision-making in wound management also identified ‘comparison’, ‘consideration’ and ‘monitoring’ through consistent evaluation. Note that these results are similar to the results obtained by this study for the assessment section. This study covers how to treat using such decision making and assessment, including flow diagrams. These findings improve practical knowledge and enhance and enrich the knowledge base for improving the performance of PI care.

When nursing a patient, nurses make many decisions. Expert nurses practice based on their know-how gained from experience, termed practical knowledge by scholars [19, 22]. Consideration of the best knowledge to use in the nursing is a field of continuing interest [27–29]. Carper said that the knowledge used by nurses is practical knowledge derived from many resources, and that it is above empirical knowledge [30]. Benner said that expert nurses use their practical experiences as a source of knowledge to apply to other cases, and that this knowledge is not only used but also further developed each time [19, 22, 31, 32]. In assessment especially, wound care nurses also make many decisions based on their empirical knowledge gained from clinical experience [33]. This is because guidelines do not consider the situations of individual patients [33]. Similarly, research has found that practice-based knowledge is the largest factor (57.5%) influencing nurses in wound care decisions [34]. Therefore, to obtain good wound care results, nurses can use their practical knowledge gained from experience to provide the appropriate ways of caring for individual patients. However, most education programs focus on guidelines and protocols. Although recently many educational programs have been augmented with hands-on lectures, they are still divided into theory and hands-on sessions, complicating the provision of integrated theoretical and practical knowledge.

In intervention, for a holistic approach to the care of chronic wounds, patient- and wound-centered concerns and follow-ups should be considered and adhered to [35]. In this study, it is said that ‘creation’ and ‘conversation’ should be managed by considering not only the wound, but also the condition of the patient and the surrounding environment, incorporating the need for integrated management, which has also been emphasized in previous studies. In addition, the framework derived in this study puts the part of ‘awareness of limitation’ at a higher level and integrates even aspects that are emphasized these days on individual competency improvement and surrounding collaboration.

Pressure ulcer care also should be managed by a holistic cycle (Fig. 1). The cognitive process for the management of PI suggested in the present study can achieve the healing of PI when the data is harmoniously and systematically collected and used together. First, the information is obtained through the ‘comparison’ step and then used in the ‘creation’ step. Next, evidence of healing is assessed in the ‘consideration’ step and a ‘conversation’ is had with the PI using the evidence. Finally, an ‘awareness of limitations’ is achieved based on the results of continuous ‘monitoring’.

A limitation of this study is that the approaches presented are sampled in only one country. In order to compensate for this, the sampling intentionally included varied participants, and the data was collected so as to achieve saturation. Also note that, the purpose of this study is to determine the nature and experiential structure of PI nursing by phenomenography rather than by obtaining a general description of the phenomenon.

The best way to improve PI management is to increase nurses’ practical competence in clinical settings. To achieve this, the practical knowledge, including perceptions of PI, proposed in this study should be thoroughly understood, with a special focus on the cognitive flow of PI management. A strength of the study was that this material provided rich data for which the participants reflected upon their lived experiences of the phenomenon of interest. The flow of the framework emphasizes to how to recognize and judge PI, rather than just theoretical PI knowledge. It effectively illuminates the actual process employed by nurses in managing PI, thereby bridging the gap between PI care knowledge and its practical application. By applying the cognitive process represented by this framework to the development of risk assessment and prevention strategies, which are currently lacking in existing guidelines [10, 11], it becomes possible to create more practical and useful guidelines. Consequently, this framework suggests a more pragmatic and valuable direction for the development of guidelines, ultimately facilitating the improvement of PI care in accordance with the nursing environment and the individual characteristics of patients.

Accordingly, this study is useful in providing new information that can be used to achieve better PI nursing. Since the findings of this study shed a light on the practical knowledge required for effective PI nursing, they can provide useful information for the development of educational programs that can be flexibly applied to not only wound care nurses but also clinical nurses and nursing students. In addition, tools or education based on a practical knowledge-based framework effectively present the varied reasoning methods used by nurses for PI management in clinical practice, thereby improving and filling a void in understanding nurse interpretation and decision-making when managing patient PI.

## Conclusion

Nurses can correctly infer how to treat PIs from the practical knowledge derived from their personal experiences. Accordingly, this study investigated the experiences of wound care nurses managing PI. Our results suggest a practical-knowledge-based frame of PI management in which assessment is conceptualized into comparison, consideration, and monitoring and intervention is conceptualized into creation, conversation, and awareness of limitations. We propose the necessity of conducting research to develop education based on the practical knowledge frame provided by this study.

## Data Availability

The datasets generated and/or analyzed during the current study are not publicly available due to [individual privacy could be compromised] but are available from the corresponding author on reasonable request.
